# A Novel Strategy for Enrichment and Isolation of Osteoprogenitor Cells from Induced Pluripotent Stem Cells Based on Surface Marker Combination

**DOI:** 10.1371/journal.pone.0099534

**Published:** 2014-06-09

**Authors:** Hiromi Ochiai-Shino, Hiroshi Kato, Takashi Sawada, Shoko Onodera, Akiko Saito, Tsuyoshi Takato, Takahiko Shibahara, Takashi Muramatsu, Toshifumi Azuma

**Affiliations:** 1 Department of Biochemistry, Tokyo Dental College, Tokyo, Japan; 2 Department of Oral and Maxillo-Facial Surgery, Tokyo Dental College, Tokyo, Japan; 3 Department of Ultrastructural Science, Tokyo Dental College, Tokyo, Japan; 4 Department of Oral and Maxillofacial Surgery, Division of Tissue Engineering, Faculty of Medicine, Graduate School of Medicine, The University of Tokyo, Tokyo, Japan; 5 Department of Endodontics and Clinical Cariology, Tokyo Dental College, Tokyo, Japan; University of Maryland, United States of America

## Abstract

In this study, we developed a new method to stimulate osteogenic differentiation in tissue-nonspecific alkaline phosphatase (TNAP)-positive cells liberated from human induced pluripotent stem cells (hiPSCs)-derived embryoid bodies (EBs) with 14 days long TGF-β/IGF-1/FGF-2 treatment. TNAP is a marker protein of osteolineage cells. We analyzed and isolated TNAP-positive and E-cadherin-negative nonepithelial cells by fluorescence-activated cell sorting. Treating the cells with a combination of transforming growth factor (TGF)-β, insulin-like growth factor (IGF)-1, and fibroblast growth factor (FGF)-2 for 14 days greatly enhanced TNAP expression and maximized expression frequency up to 77.3%. The isolated cells expressed high levels of osterix, which is an exclusive osteogenic marker. Culturing these TNAP-positive cells in osteoblast differentiation medium (OBM) led to the expression of runt-related transcription factor 2, type I collagen, bone sialoprotein, and osteocalcin (*OCN*). These cells responded to treatment with activated vitamin D3 by upregulating *OCN*. Furthermore, in OBM they were capable of generating many mineralized nodules with strong expression of receptor activator of NF-kappaB ligand and sclerostin (SOST). Real-time RT-PCR showed a significant increase in the expression of osteocyte marker genes, including *SOST*, neuropeptide Y, and reelin. Scanning electron microscopy showed dendritic morphology. Examination of semi-thin toluidine blue-stained sections showed many interconnected dendrites. Thus, TNAP-positive cells cultured in OBM may eventually become terminally differentiated osteocyte-like cells. In conclusion, treating hiPSCs-derived cells with a combination of TGF-β, IGF-1, and FGF-2 generated TNAP-positive cells at high frequency. These TNAP-positive cells had a high osteogenic potential and could terminally differentiate into osteocyte-like cells. The method described here may reveal new pathways of osteogenesis and provide a novel tool for regenerative medicine and drug development.

## Introduction

The development of new treatment strategies for osteoporosis and other skeletal tissue diseases has become increasingly important considering the growing population of elderly people. Regenerative medicine and the development of new molecular-targeted agents are aimed at providing novel tools to address these clinical demands. Induced pluripotent stem cells (iPSCs) have attracted the attention of basic and clinical researchers since their establishment because they have the potential to provide useful tools for regenerative medicine and drug development. Before the development of iPSCs, human mesenchymal stem cells (hMSCs) were promising candidates for bone engineering and regeneration, and many successful studies with these cells have been reported. However, hMSCs have several limitations. hMSCs obtained from elderly people are generally low in number, grow slowly, and show diverse differentiation potentials. Utilization of hMSCs for drug development is difficult because of their limited proliferative ability and the poor reproducibility of the method. These problems could be resolved using human iPSCs (hiPSCs). However, the osteogenic differentiation of hiPSCs presents numerous problems, including time-consuming methods, poor reproducibility, and low efficiency. The designed differentiation of hiPSCs into osteolineage cells remains difficult and impedes progress. Several reports have described the directed differentiation of iPSCs or embryonic stem cells (ESCs) into multipotent progenitors or osteoprogenitors [1], [2]. MSCs or MSC-like cells can be obtained from human ESCs by methods, such as fluorescence-activated cell sorting (FACS) after embryoid body (EB) formation. These protocols require prolonged serial passages or multiple cell sorting steps and are labor-intensive, time-consuming, and generally inefficient [3].

Other skeletal tissues, such as muscles, can also be successfully generated from hiPSCs [4]. Goudenege et al. reported that hiPSC-derived MSCs can be efficiently induced to undergo myogenic differentiation with *MYOD1* overexpression [5]. However, these protocols have low reproducibility, probably because of the heterogeneous populations of MSCs that are derived from hiPSCs. The other potential approach for generating skeletal tissues is to isolate paraxial mesodermal progenitors, which may differentiate into myogenic, osteogenic, and chondrogenic tissues [6]. Platelet-derived growth factor receptor-α-positive and KDR-negative cells are immature, and thereby can differentiate into multiple types of tissues. Platelet-derived growth factor receptor-α-positive cells are partially differentiated and can be directed to differentiate into osteolineage cells. Tanaka et al. reported that *MYOD1* overexpression in immature hiPSCs stimulates them to become mature myocytes with very high efficiency and reproducibility [7]. Their method provides relatively uniform undifferentiated cells, which may preclude variation in their differentiation frequency. Their results suggested that obtaining relatively uniform types of cells as early as possible may be very important.

We developed a new strategy to purify osteoprogenitors from EB-derived cells by isolating tissue-nonspecific alkaline phosphatase (TNAP)-positive cells using FACS. We found that cells separated from EBs did not express TNAP immediately after single-cell separation. They did not express E-cadherin but expressed relatively high levels of CD90, indicating that they were not progenitors of liver or bile duct epithelial cells. Treating the cells with a combination of transforming growth factor (TGF)-β, insulin-like growth factor (IGF)-1, and fibroblast growth factor (FGF)-2 greatly enhanced TNAP expression. Furthermore, the cells began to express high levels of osterix (OSX), which is an exclusive osteogenic marker. The cells initially expressed low levels of runt-related transcription factor 2 (RUNX2), and continuous culture induced high levels of RUNX2, bone sialoprotein (BSP), type I collagen (COL1A1), and eventually, osteocalcin (OCN). To the best of our knowledge, these are the first observations of osteoprogenitors expressing high levels of *TNAP* and *OSX* but low levels of *RUNX2* and *collagen1α*. In general, MSCs *in vivo* first express *RUNX2*, which promotes the expression of several early osteogenic marker proteins [8]. These *RUNX2*-expressing precursors then express *OSX* and induce differentiation of these cells into mature and functional osteoblasts. Therefore, OSX is a target molecule of RUNX2. However, in our experiment, OSX may have functioned as an initial transcription factor to initiate osteogenesis. We also found that these cells could form multiple mineralized nodules with multidendritic cells that express high levels of receptor activator of NF-kappaB ligand (RANKL), suggesting they can terminally differentiate into osteocyte-like cells. These cells are easily obtained from iPSCs and are capable of differentiating into osteocyte-like cells; they responded to treatment with activated vitamin D3 by upregulating OCN, providing a new clue in the investigation of osteocytes.

## Materials and Methods

### Cell culture

hiPSCs (line 201B7, Riken Cell Bank, Tsukuba, Japan) [9] were maintained with SNL76/7 feeder cells [clonally derived from a mouse fibroblast Sandoz inbred mouse-derived thioguanine-resistant and ouabain-resistant (STO) cell line transformed with neomycin resistance and murine LIF genes; American Type Culture Collection, Manassas, VA, USA] in human ES medium [Dulbecco's modified Eagle's medium: nutrient mixture F-12 (DMEM/F-12) (Invitrogen, Carlsbad, CA, USA) with 20% knockout serum replacement (Invitrogen) supplemented with 1× nonessential amino acid solution (Chemicon, Temecula, CA, USA), 2 mM l-glutamine (Chemicon), 1 mM 2-mercaptoethanol (Wako Pure Chemical Industries Ltd., Osaka, Japan), 1% penicillin/streptomycin (Invitrogen), and 5 ng/ml human FGF-2 (ReproCELL Inc., Yokohama, Japan)].

### EB formation and *in vitro* differentiation

**Figure 1:**
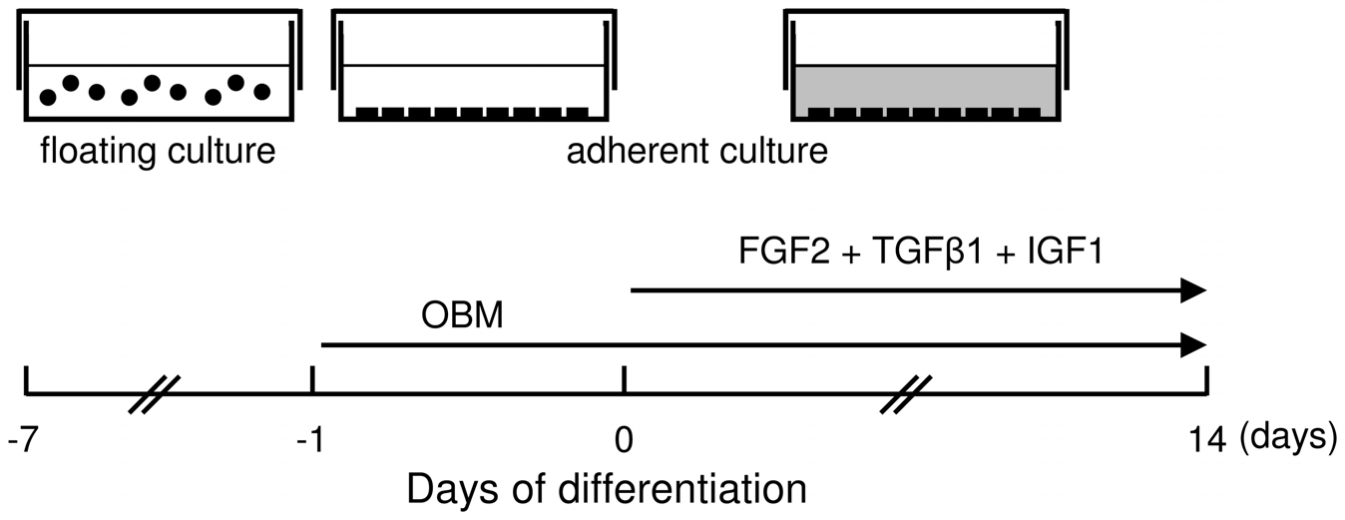
Schematic representation of the protocol for differentiation of hiPSCs into osteoblast-like cells. EBs were prepared by culturing on low-attachment Petri dishes for 6 days and dissociated in 0.5 mg/ml collagenase type IV and 0.05% trypsin–EDTA. The trypsinized EBs were cultured in OBM on cell culture dishes. Next day, various cytokines were added to the dishes (day 0) and the OBM containing cytokines was changed every 3 days. After 14 days, the cells were analyzed and isolated by FACS.

The differentiation method is shown in Figure 1. hiPSC colonies were dissociated with a cell scraper and transferred to low-attachment Petri dishes to generate EBs. EBs were maintained in suspension culture in human ES medium without FGF-2 for 6 days. EBs were then cultured in human ES medium with 2 µM thiazovivin without FGF-2 for 1 h at 37°C. After preincubation with 2 µM thiazovivin, EBs were collected and dissociated in 0.5 mg/ml collagenase type IV (Wako Pure Chemical Industries Ltd.) for 20 min at 37°C, followed by incubation in 0.05% trypsin–EDTA (Invitrogen) for 5 min at 37°C. The trypsinized EBs were seeded onto cell culture dishes at a density of 1.8×10^4^ cells/cm^2^ and cultured in osteoblast differentiation medium (OBM), which consisted of α-MEM (Invitrogen) supplemented with 10% FBS, 50 µg/ml l-ascorbic acid (Wako Pure Chemical Industries Ltd.), 10 mM β-glycerophosphate (Wako Pure Chemical Industries Ltd.), and 10 nM dexamethasone (Wako Pure Chemical Industries Ltd.). Various combinations of cytokines [25 ng/ml FGF-2, 1 ng/ml TGF-β1 (Wako Pure Chemical Industries Ltd.), 100 ng/ml IGF-1 (Wako Pure Chemical Industries Ltd.), and 50 ng/ml bone morphogenetic protein (BMP)-2/-7 (R&D Systems, Minneapolis, MN, USA)] were added on the following day (day 0) and cultured for 14 days. OBM containing fresh cytokines was resupplied every 3 days. Human periodontal ligament cells (HPDLCs; Lonza, Basal, Switzerland) were cultured in OBM for 5 days. We previously reported that HPDLCs could differentiate into osteogenic cells by culturing in OBM [10].

### Alkaline phosphatase (ALP) activity staining

Two weeks after stimulation, the cells were washed two times with phosphate-buffered saline (PBS), fixed in 4% paraformaldehyde for 5 min at room temperature, and washed three times with water. For staining, an ALP substrate solution (Roche Diagnostics, Basel, Switzerland) was added to the fixed cells for 60 min at room temperature. After staining, the cells were washed three times with distilled water, and the images were analyzed.

### Antibodies, cell staining, flow cytometric analysis, and cell sorting

After 2 weeks of osteogenic differentiation, cells from hiPSC-derived EBs that had differentiated in culture in OBM were trypsinized with 0.05% trypsin–EDTA for 10 min at 37°C. The trypsinized cells were stained with anti-human ALP phycoerythrin-conjugated antibody (R&D Systems) for 45 min on ice in the dark. After staining, the cells were washed three times with PBS, suspended in PBS containing 0.5% FBS, passed through a 40- µm mesh filter, and maintained at 4°C until flow cytometric analysis and cell sorting. Dead cells were excluded from flow cytometric analysis on the basis of propidium iodide staining (2 µg/ml) and forward scatter. We used a FACSAria (Becton-Dickinson, San Jose, CA, USA) which is a high speed cell sorter for measuring and sorting fluorescently labeled cells. Because a FACSAria is compatible with analyzing and sorting cells at the same time, we used a FACSAria to sort TNAP-positive cells. These TNAP-positive cells were found in cells cultured for 14 days in OBM supplemented with TGF-β, IGF-1, and FGF-2. After this cultivation in OBM, we sorted TNAP-positive cells by FACS.

### RNA isolation and reverse transcription gene expression

Reverse transcription-polymerase chain reaction (RT-PCR) was used to examine the expression of ALP isozymes and osteocyte markers. Real-time RT-PCR (qRT-PCR) was used to examine the expression of markers of ES cells, osteoblasts, and osteocytes. Total RNA was extracted using QIAzol reagent (Qiagen Inc., Valencia, CA, USA) according to the manufacturer's instructions. cDNA was synthesized using a high-capacity cDNA reverse transcription kit (Applied Biosystems, Foster City, CA, USA). RT-PCR was performed with GoTaq DNA polymerase (Promega, Madison, WI, USA). Target genes were germ cell-specific ALP (*GCAP*), placenta-specific ALP (*PLAP*), intestine-specific ALP (*IAP*), tissue-nonspecific ALP (*TNAP*), dentin matrix protein 1 (*DMP1*), *FGF23*, phosphate regulating endopeptidase homolog X-linked (*PHEX*), matrix extracellular phosphoglycoprotein (*MEPE*), and podoplanin (*PDPN*). Beta-actin (*β-actin*) was used as an internal control. The primers for these genes are described in Table 1. qRT-PCR was performed using the Premix Ex Taq reagent (Takara Bio Inc., Shiga, Japan) according to the manufacturer's instructions. Target genes were *OCT3/4*, *SOX2*, *NANOG*, *REX1*, *ESG1*, telomerase reverse transcriptase (*TERT*), *RUNX2*, *TNAP*, *BSP*, *COL1A1*, *OSX*, *OCN*, sclerostin (*SOST*), reelin (*RELN*), and neuropeptide Y (*NPY*). Glyceraldehyde phosphate dehydrogenase (*GAPDH*) or 18S rRNA was used as an internal control. All primers and probes are presented in Table 2 and were designed using Probefinder v2.49 (https://qpcr.probefinder.com/organism.jsp). Relative expression of genes of interest was estimated using the ΔΔ*Ct* method.

**Table 1:** Primers used for RT-PCR.

Gene symbol	Forward primer sequence	Reverse primer sequence
*GCAP*	agctcatactccatacctg	cacccccatcccgtca
*PLAP*	ctcatactccatgccca	cacccccatcccatcg
*IAP*	ctgcagccggttcctgg	gcacccccaacccatcg
*TNAP*	acatctgaccactgcca	gagacacccatcccatc
*DMP1*	caggagcacaggaaaaggag	ctggtggtatcttgggcact
*FGF23*	tatttcgacccggagaactg	ggtatgggggtgttgaagtg
*PHEX*	aagaggaccctgggagaaaa	gggactgtgagcaccaattt
*MEPE*	ccctttctgaagccagtgag	ttttcttcccccaggagttt
*PDPN*	ccagcgaagaccgctataag	acgatgattgcaccaatgaa
*beta-actin*	gggaaatcgtgcgtgacatta	ggcagtgatctccttctgcat
*Runx2*	gcccaggcgtatttcaga	tgcctggctcttcttactgag
*GAPDH*	agcttgtcatcaacgggaag	tttgatgttagtggggtctcg

**Table 2:** Primers used for qRT-PCR.

Gene symbol	GenBank accession no.	Forward primer sequence	Reverse primer sequence
			
*OCT3/4*	NM_001173531.1	caatttgccaagctcctga	agatggtcgtttggctgaat
*SOX2*	NM_003106.3	ctccgggacatgatcagc	ggtagtgctgggacatgtga
*NANOG*	NM_024865.2	atgcctcacacggagactgt	cagggctgtcctgaataagc
*REX1*	NM_174900.3	ggccttcactctagtagtgctca	ctccaggcagtagtgatctgagt
*ESG1*	NM_001025290.2	cagaggtgttccaggtccag	ctcgatgtaagggattcgaga
*TERT*	NM_001193376.1	gccttcaagagccacgtc	ccacgaactgtcgcatgt
*RUNX2*	NM_001024630.2	gtgcctaggcgcatttca	gctcttcttactgagagtggaagg
*ALP*	NM_000478.3	caaccctggggaggagac	gcattggtgttgtacgtcttg
*COL1A1*	NM_000088.3	gggattccctggacctaaag	ggaacacctcgctctcca
OSX	NM_152860.1	catctgcctggctccttg	caggggactggagccata
*OCN*	NM_199173.3	tgagagccctcacactcctc	acctttgctggactctgcac
*SOST*	NM_025237.2	agctggagaacaacaagacca	agctgtactcggacacgtctt
*RELN*	NM_005045.3	tgagagccagcctacagga	tcgttccacattctgtaccaa
*NPY*	NM_000905.3	ctcgcccgacagcatagta	gccccagtcgcttgttac
*GAPDH*	NM_002046.3	agccacatcgctcagacac	gcccaatacgaccaaatcc
18S rRNA	M11188.1	cggacaggattgacagattg	cgctccaccaactaagaacg

### Histochemistry for osteogenesis

Alizarin Red staining was performed as described previously [10]. In brief, the cultured cells were fixed with 4% paraformaldehyde in PBS for 5 min at room temperature, washed two times in PBS, incubated in Alizarin Red S solution for 5 min at room temperature, and washed five times in PBS at room temperature. Images were captured using a phase-contrast microscope.

### Immunohistochemistry

The cells were fixed with 4% paraformaldehyde in PBS for 1 h. After washing, nonspecific binding of antibodies was blocked with 5% BSA in Tris-buffered saline with 0.05% Tween 20 (TBST, pH 7.6) at room temperature for 1 h. The cells were incubated with the primary antibody in TBST containing 5% BSA for overnight at 4°C (1∶100 for goat anti-RANKL and 1∶50 for rabbit anti-SOST; both from Santa Cruz Biotechnology, Dallas, TX, USA). The secondary antibodies were fluorescein isothiocyanate-conjugated anti-goat IgG (1∶200; Santa Cruz Biotechnology) and fluorescein isothiocyanate-conjugated anti-rabbit IgG (1∶200; Zymed Laboratories, South San Francisco, CA, USA). The secondary antibodies were diluted in TBST and the cells were incubated for 1 h in the dark. The cells were finally stained with DAPI for nuclear staining.

### Electron microscopy

For scanning electron microscopy (SEM), after FACS sorting, both TNAP-positive and -negative cells were cultured in OBM for 120 days. The cells were fixed in 2.5% glutaraldehyde and 2% paraformaldehyde in 0.1 M cacodylate buffer, pH 7.4, for overnight at 4°C. After washing with 0.1 M cacodylate buffer, the samples were dehydrated in a graded series of ethanol and immersed in *t*-butyl alcohol for 30 min at 4°C. Next, the samples were freeze-dried with an ID-2 freeze dryer (Eiko, Tokyo, Japan) and sputter-coated with a cool sputter coater (SC500A, VG Microtech, East Sussex, UK). Finally, the samples were examined under a scanning electron microscope (SU-6600, Hitachi Co., Tokyo, Japan). For transmission electron microscopy (TEM), after FACS sorting, TNAP-positive cells were cultured in OBM for 120 days. The cells were fixed in 2.5% glutaraldehyde and 2% paraformaldehyde in 0.1 M cacodylate buffer, pH 7.4, for overnight at 4°C. After washing with 0.1 M cacodylate buffer, the samples were postfixed with 1% osmium tetroxide in cacodylate buffer for 1 h at room temperature. The samples were then dehydrated and embedded in epoxy resin (Epon 812, Taab, Aldermaston, UK) for thin sectioning. Semi-thin sections were stained with toluidine blue for light microscopy. Thin sections were stained with uranyl acetate and lead citrate and examined by TEM (H-7650, Hitachi Co.).

### Statistical analysis

Data are expressed as mean ± S.D. and were analyzed using ANOVA, the Bonferroni test, or Student's *t*-test. All data are representative of at least three independent experiments. Statistical significance was defined as *p*<0.05.

## Results

### TNAP-positive cells derived from human iPSCs

**Figure 2:**
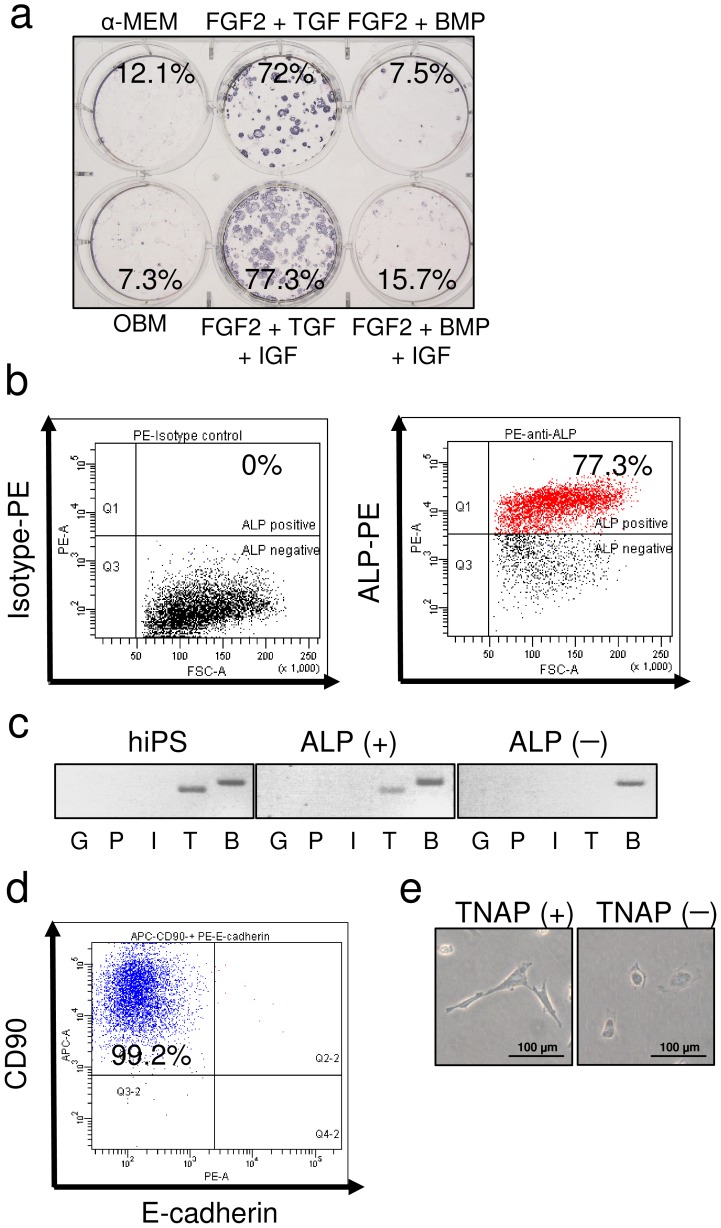
Characterization of ALP-positive cells derived from human iPSCs. (a) Single cells from hEBs were cultured with various cytokines for 2 weeks and stained for ALP activity. The cells were cultured in α-MEM containing 10% FBS (α-MEM); α-MEM containing 10% FBS, ascorbic acid, and β-glycerophosphate (β-GP) (OBM); OBM with FGF-2 and TGF-β1 (FGF2 + TGF); OBM with FGF-2, TGF-β1, and IGF-1 (FGF2 + TGF + IGF); OBM with FGF-2 and BMP-2/-7 (FGF2 + BMP); or OBM with FGF-2, BMP-2/-7, and IGF-1 (FGF2 + BMP + IGF). The percentages shown indicate the frequency of ALP-positive cells determined by FACS analysis. (b) FACS analysis for the isolation of ALP-positive cells (right) and isotype control (left). (c) Expression of ALP isoenzymes: germ cell-specific ALP (G), placenta-specific ALP (P), intestine-specific ALP (I), tissue-nonspecific ALP (T), and β-actin (B) in parental hiPSCs, isolated ALP-positive cells, and isolated ALP-negative cells. (d) FACS analysis of CD90 and E-cadherin in the TNAP-positive population. (e) Morphology of TNAP-positive (TNAP+) and TNAP-negative (TNAP−) cells.

We induced EBs from hiPSCs and then the trypsinized EBs. TNAP-positive cells were not observed immediately after trypsinization. The trypsinized EBs were cultured in OBM with various cytokines. We investigated which combination of cytokines was most effective in inducing TNAP-positive cells. The number of TNAP-positive cells was markedly increased by treatment with a combination of FGF-2, TGF-β, and IGF-1, and these cells could be isolated using FACSAria (Fig. 2a, b). ALP isoenzymes are categorized into four classes: germ cell specific, placenta specific, intestine specific, and tissue nonspecific. Parental hiPSCs and ALP-positive cells derived from hiPSCs by EB formation expressed the TNAP isoenzyme, whereas ALP-negative cells did not express any ALP isoenzyme (Fig. 2c). Subsequently, TNAP-positive cells were assessed for the expression of the MSC marker CD90 and the epithelial cell marker E-cadherin. Almost all cells were also CD90-positive and E-cadherin-negative (Fig. 2d). As shown in Figure 2e, TNAP-positive cells were spindle shaped, like MSCs, and TNAP-negative cells were cuboidal, like epithelial cells. These data suggested that TNAP-positive cells derived from hiPSCs were not epithelial cells.

### TNAP-positive cells expressed various osteoblast marker genes

**Figure 3:**
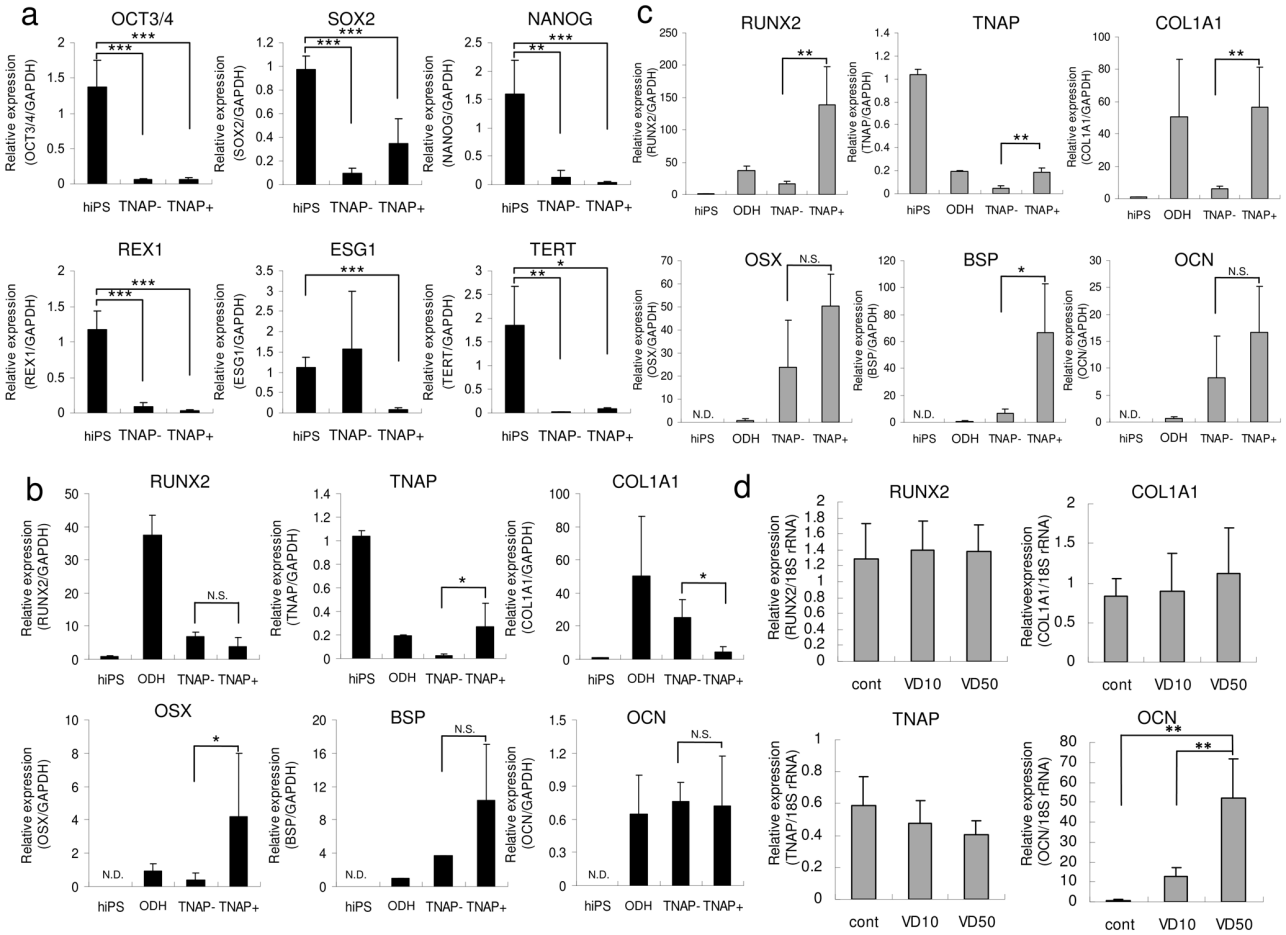
Characterization of gene expression in TNAP-positive iPSCs. (a) Comparison of expression of ES cell markers in TNAP-negative (TNAP−) and TNAP-positive (TNAP+) cells. qRT-PCR analysis of *OCT3/4*, *SOX2*, *NANOG*, *REX1*, *ESG1*, and *TERT* was performed in isolated cells using a FACSAria cell sorter. Parental iPSCs (hiPS) was used as a positive control. (b) Comparison of the expression of markers of osteoblast differentiation in TNAP-negative (TNAP−) and TNAP-positive (TNAP+) cells. qRT-PCR analysis was performed in cells isolated by FACS. Parental iPSCs (hiPS) were used as a negative control and osteogenic-differentiated HPDLCs (ODH) as a positive control. (c) qRT-PCR analysis was performed with cells grown in OBM for 40 days. (d) After isolation by FACS, TNAP-positive cells were treated with vehicle (cont), 10 nM active vitamin D3 (VD10), or 50 nM active vitamin D3 (VD50) for 6 days. Abbreviations: RUNX2, runt-related transcription factor 2; TNAP, tissue-nonspecific alkaline phosphatase; COL1A1, type I collagen; OSX, osterix; BSP, bone sialoprotein; OCN, osteocalcin. The expression of these genes was analyzed by qRT-PCR, and the mRNA levels of the genes were normalized to that of *GAPDH* or 18S rRNA. The experiments were performed in triplicate. Values represent mean ± S.D. (*n*  =  4). *p<0.05, **p<0.01, ***p<0.005.

We determined the expression of ES cell markers and osteoblast-specific markers to confirm osteoblast differentiation. The expression of ES cell markers *OCT3/4*, *SOX2*, *NANOG*, *REX1*, *ESG1* (*DPPA5*), and *TERT* was markedly reduced in both TNAP-positive and -negative cells compared with hiPSCs (Fig. 3a). In TNAP-positive cells, we found marked expression of not only *TNAP* but also *OSX* (Fig. 3b). Although the expression of *RUNX2* and *COL1A1* was relatively low, the expression of the marker for committed osteoblasts *OSX* was markedly increased, suggesting that these cells were osteolineage cells. Compared with TNAP-negative cells, the expression of the osteoblast-specific markers *TNAP*, *OSX*, *BSP*, and *OCN* was markedly upregulated in TNAP-positive cells cultured in OBM for 40 days (Fig. 3c). The expression of osteoblast markers *RUNX2*, *COL1A1*, and *BSP* was markedly increased in TNAP-positive cells when these cells were cultured for 40 days. In addition, *OCN* expression showed a significant increase and *TNAP* expression showed a decreasing trend with vitamin D3 treatment (Fig. 3d). These observations indicated that these cells could respond to osteogenic reagents and could differentiate into cells in the late phase of osteogenesis.

### TNAP-positive cells expressed characteristics of osteocyte-like cells

**Figure 4:**
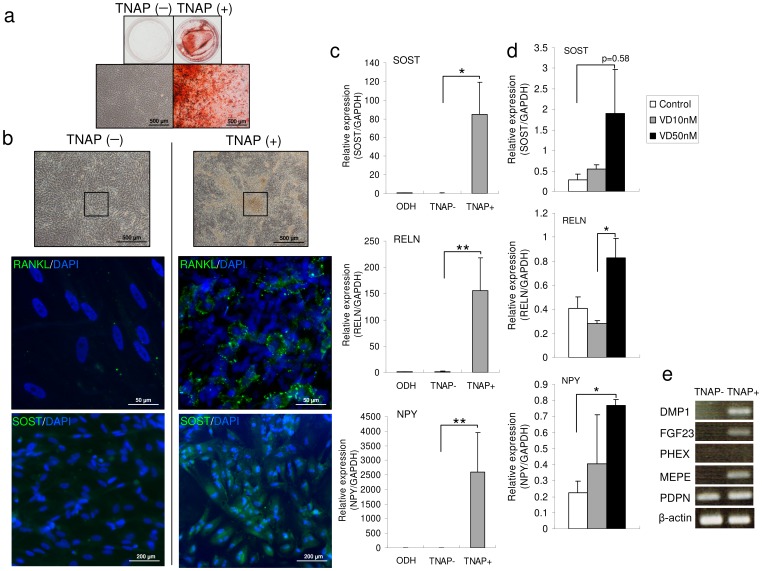
TNAP-positive cells express various osteocyte markers. (a) Osteogenic differentiation was confirmed by Alizarin Red staining after 40 days in OBM. The upper panels are whole-well images and lower panels are magnified images. (b) Phase-contrast images of TNAP-negative and -positive cells derived from hiPSCs at day 40 of culture in OBM (upper panel). The black box in the upper images represents the region shown in the middle and lower images. (c) Comparison of the expression of osteocyte marker genes between TNAP-negative (TNAP−) and TNAP-positive (TNAP+) cells by qRT-PCR. Osteogenic-differentiated HPDLCs (ODH) were used as a control. (d) TNAP-positive cells were treated with vehicle (white bars), 10 nM vitamin D3 (gray bars), or 50 nM vitamin D3 (black bars) for 6 days. (e) Comparison of expression of osteocyte marker genes between TNAP-negative (TNAP−) and TNAP-positive (TNAP+) cells by RT-PCR. Abbreviations: SOST, sclerostin; RELN, reelin; NPY, neuropeptide Y. Expression of these genes was analyzed by qRT-PCR, and mRNA levels of the genes were normalized to that of *GAPDH*. The experiments were performed in triplicate. Values represent mean ± S.D. (*n*  =  4). *p<0.05, **p<0.01.

After culture in OBM for 40 days, TNAP-positive cells differentiated into osteocyte-like cells containing an abundant calcium matrix, as revealed by Alizarin Red-positive staining in the well (Fig. 4a). In contrast, TNAP-negative cells exhibited no potential to form calcium-positive osteocytes, as indicated by the absence of Alizarin Red staining (Fig. 4a). Many mineralized nodule-like structures were observed in cultures of TNAP-positive cells but not in those of TNAP-negative cells. In addition, TNAP-positive cells expressed RANKL in the areas of mineralized nodule-like structures. The expression of SOST was observed only in TNAP-positive cells but not in TNAP-negative cells (Fig. 4b). The expression of osteocyte markers *SOST*, *RELN*, and *NPY* was significantly increased in TNAP-positive cells (Fig. 4c). The expression of these osteocyte markers increased concentration-dependently with vitamin D3 administration only after 6 days in culture (Fig. 4d). As shown in Figure 4e, expression of the osteocyte marker genes *DMP1*, *FGF23*, and *MEPE* was detected in TNAP-positive cells.

### Morphology of osteocyte-like cells as observed by electron microscopy

**Figure 5:**
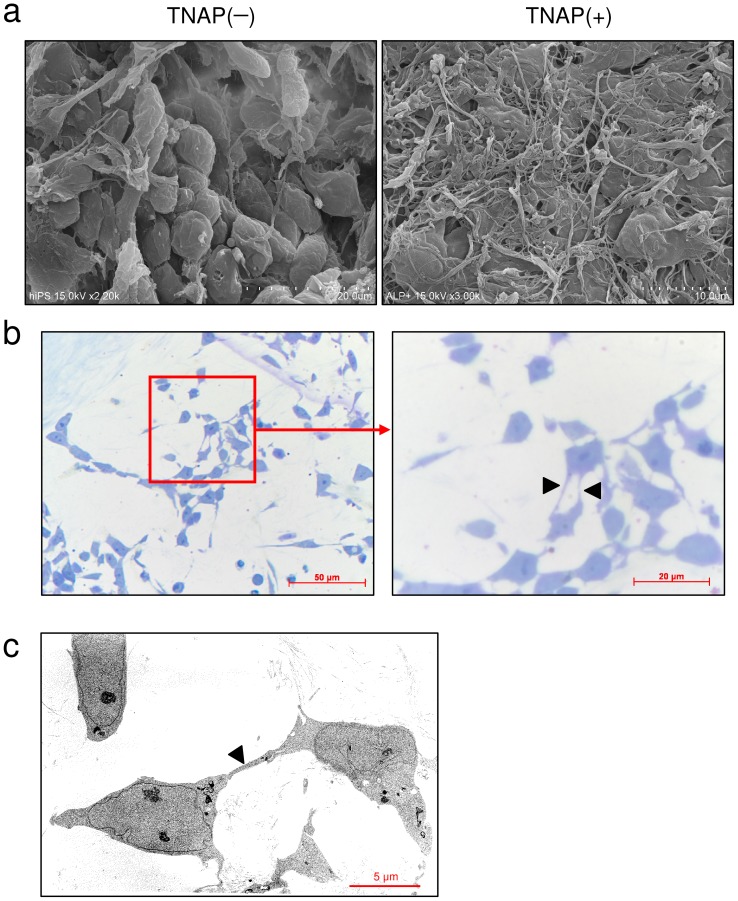
SEM and TEM images of TNAP-positive cells induced to differentiate into osteocyte-like cells. After isolation by FACS, TNAP-positive and -negative cells were cultured in OBM for 120 days. (a) SEM images of TNAP-negative (TNAP−) and TNAP-positive (TNAP+) cells. (b) Images of toluidine blue-stained semi-thin sections of TNAP-positive cells are shown at low (left) and high (right) magnification with light microscopy. (c) TEM image of TNAP-positive cells. Arrowheads indicate cytoplasmic processes.

After culture in OBM for 120 days, many cytoplasmic processes were observed in TNAP-positive cells (Fig. 5a). In contrast, TNAP-negative cells were round and had no cytoplasmic processes (Fig. 5a). Cell–cell contact with a cytoplasmic process was observed in TNAP-positive cells (Fig. 5b, c, arrowheads).

## Discussion

iPSCs are powerful tools in many fields of basic scientific research. Several reports have shown that osteogenic cells can be generated from iPSCs [3],[6],[7],[11]–[15]. The reported methods for the generation of osteogenic cells are time-consuming and labor-intensive and include repeated passages to select fast-growing adhesive cells [2]. The phenotypic characteristics of these cells are similar to those of mesenchymal cells. Bilousova et al. [14] reported that retinoic acid treatment of murine iPSCs cultured in OBM for several weeks resulted in cells that were positive for osteogenic markers and Alizarin Red staining. This so-called outgrowth method essentially requires no supplements other than OBM. However, human iPSCs are not as simple to differentiate as murine iPSCs. Multistep, labor-intensive processes are often necessary. Mahmood et al. [16] reported that iPSCs that were cultured in low-adhesive plastic Petri dishes with the TGF-β inhibitor SB-431542 for 10 days formed EBs and adhered to the cell culture dishes. These cells could be passaged 4–11 times. The cells were then transferred into OBM and cultured for an additional 20 days, eventually forming osteoblasts. Villa-Diaz et al. [3] used synthetic polymer-coated dishes to generate MSCs. It is possible that these MSCs derived from iPSCs were a mixed population of cells, although the protocol usually requires a long period of time. Thus, methods that are simpler and less time-consuming are desired.

The most essential proteins for mineralization by osteolineage cells are COL1A1 and ALP [17]. Humans have four ALP genes encoding intestinal, placental, placenta-like, and liver/bone/kidney (i.e., TNAP) gene products. TNAP is localized on the outside of the plasma membrane of cells and in the membrane of matrix vesicles and is attached to the membrane by a glycophosphatidylinositol anchor. TNAP in osteolineage cells is expressed relatively early during differentiation and is abundantly expressed on the membrane surface. Apart from osteolineage cells, epithelial cells also express TNAP. Therefore, accumulation of relatively pure osteolineage cells among TNAP-positive cells will probably require elimination of epithelial cells. Some cells among EBs derived from hiPSCs expressed ALP (Fig. 2a, b). RT-PCR clearly showed that these cells expressed TNAP (Fig. 2c). Morphologically, TNAP-positive cells were fibroblastic and spindle shaped rather than cuboidal or epithelial shaped (Fig. 2e). We next examined whether these cells expressed E-cadherin. Most cells were E-cadherin-negative and CD90-positive, indicating that TNAP-positive cells are probably not epithelial cells. We further investigated methods of maximizing TNAP expression. Previous reports indicated that activins, retinoic acids, and BMPs are capable of inducing osteolineage cells from ESCs or iPSCs [6], [13], [14]. We attempted several combinations of cytokines and found that activins, retinoic acids, and BMPs did not effectively induce TNAP expression (data not shown). However, exposure to TGF-β, IGF-1, and FGF-2 in OBM had the most potent TNAP-inducing effects (Fig. 2a). Although the mechanisms by which ALP expression is regulated are complex, the BMP/Runx2 and Osx systems are believed to be the principal regulatory pathways controlling osteoblast differentiation and TNAP expression [8]. In brief, the BMP/Smad pathway targets activation of Runx2, which in turn activates Osx expression. Because epigenetic conditions during embryonic development are quite different from those during ESC/iPSC differentiation, the transcription factors required for TNAP expression may be different. In embryos, Runx2 is required for the differentiation of prechondrogenic mesenchymal cells into osteoblasts, whereas Osx is believed to induce subsequent maturation of osteoblasts and inhibit chondrogenic differentiation. In Osx-null embryos, cartilage forms normally but the embryos completely lack bone [17]–[19]. *OSX*, which is specifically and exclusively expressed in all osteoblasts, showed markedly high expression in TNAP-positive cells, although TNAP-positive and -negative cells expressed almost similar levels of *RUNX2* (Fig. 3b). These findings indicated that iPSCs may not require the prechondrogenic process and may induce Osx without a Runx2 surge. Several pathways have been reported to increase Osx expression. Mitogen-activated protein kinases, particularly p38, Erk1/2, and protein kinase D, activate Osx expression accompanied by TNAP activation. Ascorbate-dependent prolyl hydroxylase domain protein induces Osx expression. Endoplasmic reticulum stress also increases Osx induction [20]. These cascades may play an important role in the Osx surge and the increase in TNAP in iPSCs. We found that continuous culture of these TNAP-positive cells in OBM eventually led to increased expression of *RUNX2*, *TNAP*, *COL1A1*, and *OSX* as well as other osteogenic markers, such as *BSP* and *OCN*. These results indicated that TNAP-positive cells derived from hiPSCs are OSX-positive osteoprogenitors, not chondrogenic cells. Furthermore, TNAP-positive cells are capable not only of differentiating into osteogenic cells but also of responding to active vitamin D treatment. Vitamin D treatment effectively upregulated *OCN* and downregulated *TNAP*, indicating that these cells could differentiate into cells in the late phase of osteogenesis and may be able to differentiate into terminally differentiated osteocytes.

Previous reports showed that murine and human ESCs cultured in OBM formed a number of bone/mineralized nodules with intense mineralization [21], [22]. A bone nodule is a group of cells with three-dimensional multistratified structures. We found that TNAP-positive cells derived from hiPSCs formed several bone nodules that contained intensely stained anti-RANKL-immunopositive cells (Fig. 4b). We also observed anti-SOST positivity in these areas (Fig. 4b). qRT-PCR and RT-PCR clearly showed a significant increase in the expression of osteocyte marker genes, including *SOST*, *NPY*, *RELN*, *DMP1*, *FGF23*, and *MEPE* (Fig. 4c, d, and e). SEM showed that TNAP-negative cells had a cuboidal morphology without dendritic structures, whereas TNAP-positive cells were flattened with multiple dendritic morphologies after cultivation in OBM (Fig. 5a). Toluidine blue-stained semi-thin sections clearly showed that these dendrites were connected to each other. The osteocyte-like cell line MLO-Y4 shows similar morphology [23]. Thus, these cells were osteocyte-like cells.

In the present study, formation of bone nodules was observed much more frequently in iPSCs than in MSCs, similar to the findings of a previous study [24]. Because ESCs and iPSCs have a higher proliferative potential, they may form multistratified structures more readily and thus provide cells with a three-dimensional microenvironment that promotes terminal differentiation into osteocyte-like cells. Although our cells were positive for most osteocyte marker proteins, the expression of *PHEX* was not increased. This difference may be related to the *in vivo* microenvironment.

In conclusion, treating trypsinized single cells with a combination of TGF-β, IGF-1, and FGF-2 generated TNAP-positive cells at a high frequency. These TNAP-positive cells had high osteogenic potential and could terminally differentiate into osteocyte-like cells. They responded to osteogenic reagents and may be a useful tool for drug evaluation.
